# Phylogenetic congruence of *Plasmodium* spp. and wild ungulate hosts in the Peruvian Amazon

**DOI:** 10.1016/j.meegid.2024.105554

**Published:** 2024-01-19

**Authors:** Gabriela M. Ulloa, Alex D. Greenwood, Omar E. Cornejo, Frederico Ozanan Barros Monteiro, Alessandra Scofield, Meddly L. Santolalla Robles, Andres G. Lescano, Pedro Mayor

**Affiliations:** a Departament de Sanitat i d’Anatomia Animals, Facultat de Veterinària, Universitat Autònoma de Barcelona, Edifici V, Bellaterra-Barcelona E-08193, Spain; b Programa de Pós-Graduação em Saúde e Produção Animal na Amazônia, Universidade Federal Rural da Amazônia (UFRA), Av. Presidente Tancredo Neves 2501, Terra Firme, Belém 66077-830, Pará, Brazil; c Grupo de Enfermedades Infecciosas Re-Emergentes, Universidad Científica del Sur (UCSUR), Lima, Peru; d Leibniz-Institute for Zoo and Wildlife Research, Alfred-Kowalke-Strasse 17, Berlin 10315, Germany; e School of Veterinary Medicine, Freie Universität Berlin, Oertzenweg 19b, 14163, Germany; f Department of Ecology and Evolutionary Biology, University of California Santa Cruz, Santa Cruz, CA, United States of America; g Laboratory of Animal Parasitology, Postgraduate Program in Animal Health in the Amazon, Institute of Veterinary Medicine, Federal University of Pará, Castanhal, Brazil; h Emerge, Research Unit on Emerging Diseases and Climate Change, Universidad Peruana Cayetano Heredia, Lima, Peru; i Comunidad de Manejo de Fauna Silvestre en la Amazonía y en Latinoamérica (COMFAUNA), 332 Malecon Tarapaca, Iquitos, Peru; j Museo de Culturas Indígenas Amazónicas, Loreto, Iquitos, Peru

**Keywords:** Brocket deer, Endemic parasites, Subsistence hunting, *Plasmodium*, Co-evolution

## Abstract

Malaria parasites are known to infect a variety of vertebrate hosts, including ungulates. However, ungulates of Amazonia have not been investigated. We report for the first time, the presence of parasite lineages closely related to *Plasmodium odocoilei* clade 1 and clade 2 in free-ranging South American red-brocket deer (*Mazama americana*; 44.4%, 4/9) and gray-brocket deer (*Mazama nemorivaga*; 50.0%, 1/2). We performed PCR-based analysis of blood samples from 47 ungulates of five different species collected during subsistence hunting by an indigenous community in the Peruvian Amazon. We detected *Plasmodium malariae/brasilianum* lineage in a sample from red-brocket deer. However, no parasite DNA was detected in collared peccary *(Pecari tajacu*; 0.0%, 0/10), white-lipped peccary *(Tayassu pecari*; 0.0%, 0/15), and tapir (*Tapirus terrestris*; 0.0%, 0/11). Concordant phylogenetic analyses suggested a possible co-evolutionary relationship between the *Plasmodium* lineages found in American deer and their hosts.

## 1. Introduction

The broad genus *Plasmodium* infects diverse animal taxa, from birds, reptiles and mammals, with human and non-human primates being the most studied hosts (Perkins and Schaer, 2016). Limited efforts worldwide have reported that ungulates are associated with parasites of the genus *Plasmodium* (Asada et al., 2018; Boundenga et al., 2016; Kaewthamasorn et al., 2018; Martinsen et al., 2016; Templeton et al., 2016a). The only reported molecular sequences of parasites in ungulates have been tentatively named based on the few morphological descriptions reported for many decades (Templeton et al., 2016b), including: a) the multiple lineages of *Plasmodium odocoilei* are closely related and are grouped into two clades (clade 1 and clade 2), found in white-tailed deer (*Odocoileus virginianus*) in different locations in the United States (Guggisberg et al., 2018; Martinsen et al., 2016); b) *Plasmodium bubalis* lineages, termed type I and II, from water buffalo (*Bubalis bubalis*) in Thailand and Vietnam (Templeton et al., 2016a); and c) *Plasmodium caprae*, a lineage from a Zambian goat (Kaewthamasorn et al., 2018).

The only study of malaria-associated ungulate parasites from South American reported *Plasmodium* lineages related to *P. odocoilei* clade 2 found in wild populations of the pampas deer (*Ozotoceros bezoarticus*) in the Brazilian Pantanal (Asada et al., 2018). It is likely that the expansion of these ungulate parasite lineages coincided with the radiation of ungulates in the Southern hemisphere (Duarte et al., 2008). Population level molecular studies in South American ungulates could help clarify the evolutionary history malarial parasites in the Americas. This is particularly true in the Amazon, a complex ecosystem where multiple hosts of malaria-associated parasites coexist in wildlife and live in close proximity to human populations in malaria-endemic areas (de Abreu et al., 2019; Amir et al., 2018; Perkins and Schaer, 2016; Voinson et al., 2022).

We detected the *Plasmodium* spp. infection in wild populations of ungulates in a nearly pristine, isolated and malaria-endemic geographical area in the Peruvian Amazon, using molecular approaches to detect the mitochondrial DNA (mtDNA) of *Plasmodium* species. It is important to highlight that the study of malaria parasites in wild populations is beset by logistical and financial restrictions, resulting in incomplete information regarding the diversity of their vertebrate hosts and environmental influences on their prevalence and distribution. Considering that subsistence hunting is a cultural activity and an important source of dietary protein for indigenous and non-indigenous populations throughout the Amazon (Jacob et al., 2023), we took advantage of blood samples collected from ungulates under sustainable hunting conditions by local indigenous communities. This methodology is appropriate to the geographical and sociocultural context of the study area and collaborations with the local population were highly advantageous to overcoming common limitations of field work in the region (Aysanoa et al., 2017; Cáceres et al., 2017; González-Olvera et al., 2022; Mayor et al., 2017).

## 2. Material and methods

### 2.1. Study area

**Fig. 1.**
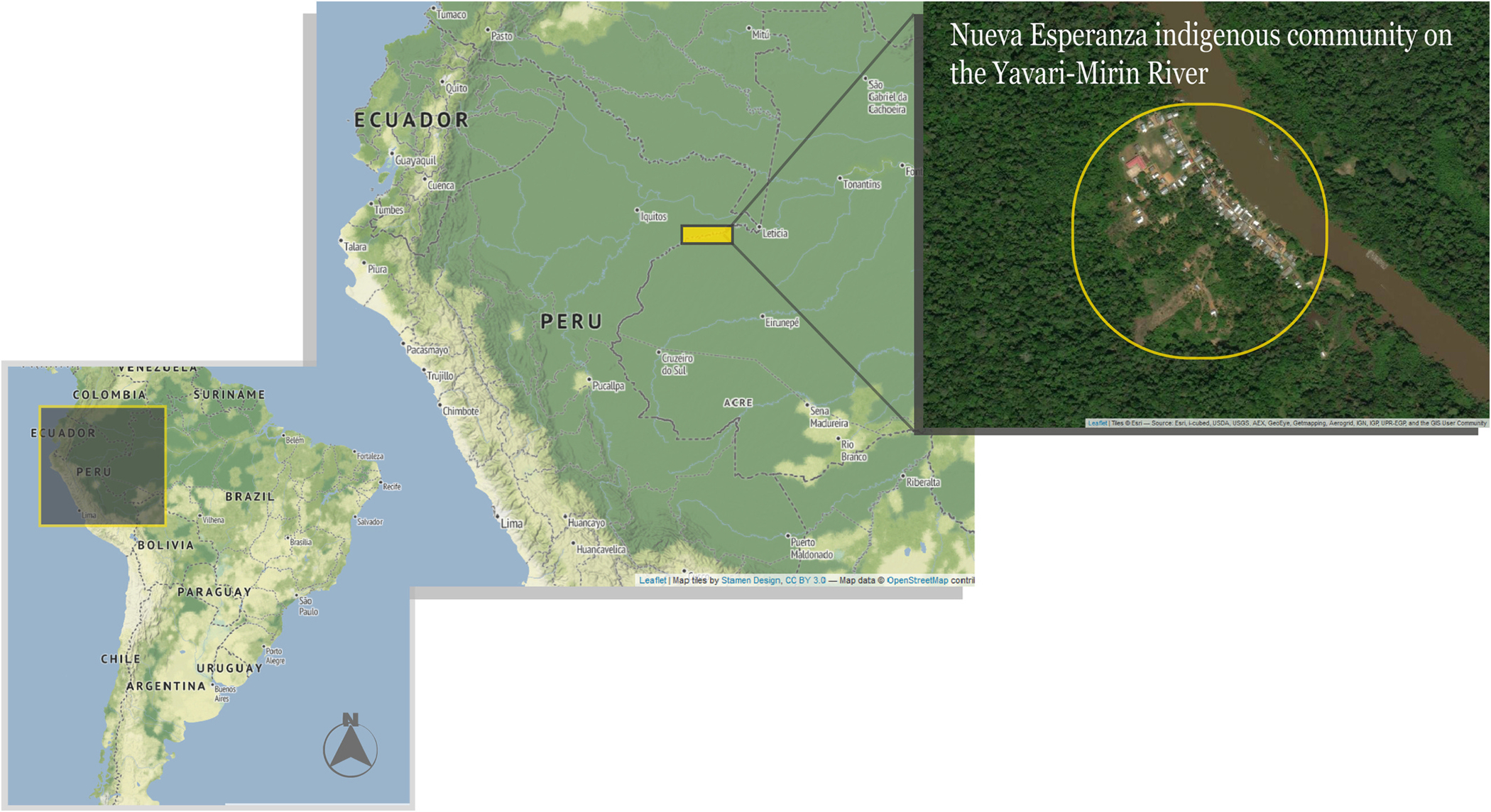
Map of study area, Nueva Esperanza indigenous community located on the slopes of the Yavari-Mirin River.

Samples were taken from an area covering 107,000 ha of contiguous, predominantly upland forest along the Yavari-Mirin River in the northeastern Peruvian Amazon (Fig. 1). There is only one human community within the study area, the indigenous Yagua community of Nueva Esperanza (04°19′53”S; 71°57′33”W; UT-5:00), with a population of 343 inhabitants in 2020 (167 males and 176 females). The Yavari-Mirin River basin is 302 km from the nearest city, Iquitos, and has the highest wildlife diversity observed in the Peruvian Amazon, especially mammals, including 14 species of non-human primates. The Peruvian Ministry of Health currently considers this region a low but stable malaria endemic area (Bernárdez-Rodriguez et al., 2021). The villagers depend on subsistence activities (hunting, fishing, other forestry activities, and small-scale agriculture), but opportunistically trade wood, fish, wild meat, and agricultural products.

### 2.2. Samples

Local hunters were trained to collect blood samples from all species hunted for subsistence purposes using discarded material from legal hunting. From 2019 to 2020, blood samples were collected from 47 ungulates belonging to five species: red-brocket deer (*Mazama americana, n* = 9), gray-brocket deer (*Mazama nemorivaga, n* = 2), white-lipped peccary (*Tayassu pecari*, *n* = 15), tapir (*Tapirus terrestris, n* = 11), and collared peccary (*Pecari tajacu, n* = 10). Hunters impregnated FTA^®^ cards (Scheilcher & Schuell, Germany) with blood from the cranial or caudal vena cavaduring postmortem processing of each animal. Local hunters were also trained to record the date, species, and sex of all animals of all taxa hunted as part of their normal subsistence activities. There was no incentive or payment to encourage additional hunting of wildlife. This sampling strategy was integrated into a community participatory program to improve the conservation and sustainability of natural resources and indigenous livelihoods (González-Olvera et al., 2022). This approach allows for the determination of wildlife population health status without promoting hunting beyond subsistence levels.

Collected biological material was sealed in individual plastic bags with desiccant and stored in the collection area at room temperature for a minimum of two weeks and a maximum of six months before being transferred to −70 °C for preservation. DNA was extracted from blood spots on filter paper using the AllPrep DNA/RNA Mini Kit (Qiagen, Germany) according to the manufacturer’s instructions at the Malaria Laboratory of the Institute of Global Health, Barcelona. The DNA was used for malaria parasite detection by nested PCR amplification, and the products were sequenced for a portion of the *Plasmodium cytb* gene.

### 2.3. Ethical review

The research protocols for the sampling of wild animals were approved by the Peruvian Forest and Wildlife Service (N° 258–2019-MINAGRI-SERFOR-DGGSPFFS) and the Institutional Animal Use Ethics Committee of the Universidad Peruana Cayetano Heredia (ref. 102,142). Dried wildlife blood samples on filter paper were exported from Peru to Spain with the approval of the Ministry of Agriculture and Irrigation (MINAGRI) through the Peruvian Forestry and Wildlife Service - SERFOR (N° 003258/SP, N° 003260/SP, N° 003568-SERFOR, N° 003579-SERFOR) according to the Nagoya Protocol.

### 2.4. Amplification of Plasmodium sequences by polymerase Chain reaction (PCR)

Ungulates blood spots samples on filter paper were screened for *Plasmodium cytb* sequences (~776 bp) as previously described (Martinsen et al., 2006), with some modifications to the PCR program conditions, as these were samples with low-density, submicroscopic infections (Guggisberg et al., 2018; Martinsen et al., 2016). Nested PCR was performed using DW2 [5 μM] 5-TAA TGC CTA GAC GTA TTC CTG ATT ATC CAG-3 and DW4 [5 μM] 5-TGT TTG CTT GGG AGC TGT AAT CAT AAT GTG-3 in the first round followed by genus-specific primers: FP3 [5 μM] 5-TAT ATA ACT TAT TTT TTG ATA TG-3 and RP3 [5 μM] 5-GTR ATW GCA TTA TCT GGA TGT GA-3 in the second round of PCR (Martinsen et al., 2006). For the first round, 5 μL of genomic DNA and 10 μL of 2× Platinum II Hot-Start PCR Master Mix (Invitrogen, USA) were used in a 20 μL reaction volume. Cycling conditions included an initial denaturation step of 94 °C for 3 min, followed by 40 cycles of denaturation (20 s at 94 °C), annealing (30 s at 62 °C), and elongation 1 min at 72 °C, followed by a final elongation step of 10 min at 72 °C. For the second PCR round, 2 μL of the primary PCR product was diluted 1/20 in DNAase and RNAase free water and used as template for nested PCR with 10 μL of 2× Platinum II Hot-Start PCR Master Mix (Invitrogen, USA) in a 20 μL reaction volume. Cycling conditions included an initial denaturation step of 3 min at 94 °C, followed by 40 cycles of denaturation (94 °C, 20 s), annealing (52 °C, 30 s) and elongation (72 °C, 1 min), followed by a final elongation step of 10 min at 72 °C. Amplified products were gel-purified using the QIA quick Gel Extraction Kit (Qiagen, Germany) and sequenced by Macrogen (Korea) to confirm *Plasmodium* infection using FP3 and RP3 primers (Protocol published in: https://www.protocols.io/private/6653CE1D8DD611ECA1850A58A9FEAC02).

Positive controls for *Plasmodium* spp. were performed on DNA extracted from *P. falciparum* strain 3D7, and molecular grade water was included as a contamination control. The second round of PCR (nPCR) was performed in a separate laboratory from the first round. The bench, instruments and materials were disinfected with DNAZap (Invitrogen) after each PCR analysis. No pipettes or racks were shared between the two rounds of PCRs.

### 2.5. Phylogenetic analysis

The chromatograms for each sequence were corrected for errors associated with saturation at the beginning of the sequences and low signal at the ends. For amplicons which were weak, duplicates were sequenced and the forward and reverse direction sequences were assembled using the Geneious Prime software (v. 2023.0.4. Biomatters Ltd.)(Kearse et al., 2012). Multiple sequence alignments were carried out using the CLUSTAL-OMEGA algorithm implemented in Geneious Prime (Thompson et al., 1994). We used different models of sequence evolution, which had the best fit to our dataset as inferred from the Akaike Information Criterion (AIC) value calculated in MEGA (v. 11.0.13). Phylogenetic relationships were inferred using maximum likelihood (ML) methods using FastTree 2.1.11(Price et al., 2009) and PhyML (Guindon et al., 2010). Nodal support was assessed by bootstrap using 2000 pseudo-replicates. Additionally, Bayesian inference (BI) using the program MrBayes 3.2.6 (Huelsenbeck and Ronquist, 2001) implemented in Geneious Prime was also used to reconstruct phylogenetic relationships. Parameters for the evolutionary model were the same as in ML and the search was carried out in two simultaneous runs of one million generations, sampled each 1000 generations, with a burn-in of 25%.

The Bayesian Markov Chain Monte Carlo (MCMC) method implemented in the BEAST v2.7.4 package was used for divergence time calculations, with a molecular clock based on the mutation rate used for *Plasmodium* mtDNA of 1.5% per million years (Pacheco et al., 2011; Silva et al., 2011). General Time Reversal (GTR) was used as the initial evolutionary model, and an uncorrelated relaxed clock model. To follow one of the assumptions of the birth-death model, only one haplotype per lineage was allowed for molecular clock analysis to avoid overestimation of divergence times. A homologous sequence from *Theileria taurotragi* (ref. NC053926.1) was used as an outgroup and calibrated against the estimated time of divergence of the human malaria parasite *P. malariae* (~23 MYA) (Pacheco et al., 2011). All divergence estimates recovered with support values greater than 0.92 in all major branches.

Phylogenetic analyses were based on ~776 bp of *cytb* sequences. We additionally analyzed 14 complete or nearly complete *cytb* genes, obtained from the NCBI nucleotide database) of: *P. odocoilei* strains obtained from samples of *Anopheles punctipennis* (accession number, KU133755.1 and KU133748.1), *P. odocoilei* strains recovered from white-tailed deer from USA (accession numbers, MG709249.1, KU133750.1, MG709243.1, MG709250.1, KU133754.1 and MH177860.1) and a *P. odocoilei* strain BRDeer-89 from Brazil (accession numbers, LC326034.1). In addition, we added existing sequences from *Plasmodium* species that cause malaria in ungulates. These include: *Plasmodium bubalis* strains of water buffalo (*B. bubalis*) from Thailand (accession number, LC090213.1 and OL672209.1) and *Plasmodium caprae* strain of Zambian goat (accession number, LC090215.1). Two *Plasmodium* species of humans and non-human primates, *P. malariae* (accession number, AB489194.1) and *P. brasilianum* strain Bolivian I (accession number, CM043783.1). Reference genome sequence and annotation files were downloaded from GenBank.

## 3. Results

The overall frequency of *Plasmodium* spp. in ungulates in the study area was 12.8% (6/47). We detected *Plasmodium* spp. lineages in the red-brocket deer (55.6%, 5/9) and the gray-brocket deer (50,0%, 1/2). However, *Plasmodium* spp. infection was not detected from the collared peccary (0/10), the white-lipped peccary (0/15) or the tapir (0/11).

**Fig. 2.**
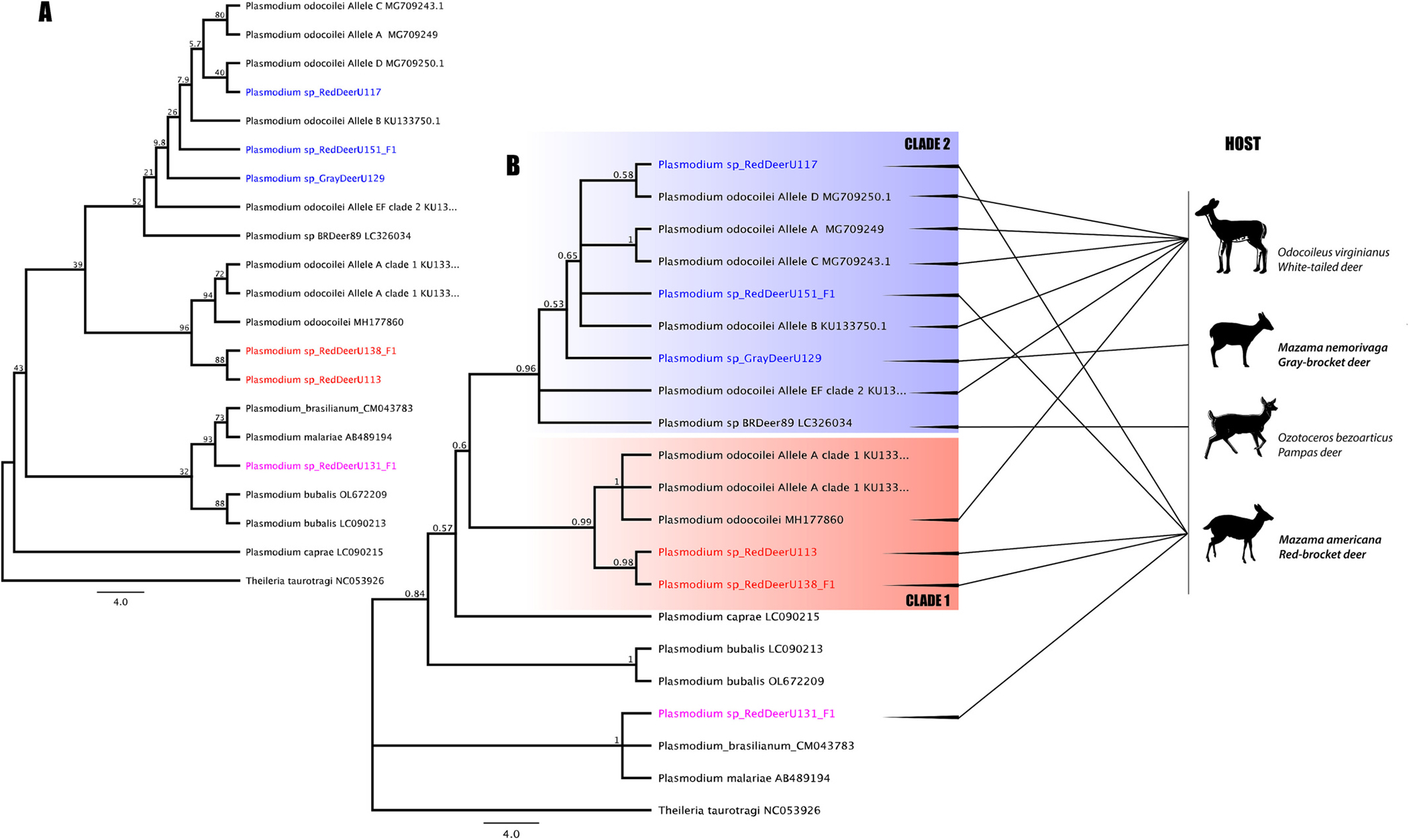
Phylogeny of Amazonian ungulate *Plasmodium* lineages. (A) Maximum likelihood (ML) tree for all ungulate *Plasmodium* lineages studied, obtained using PhyML with 2000 replicates. (B). Bayesian inference (BI) tree obtained using MrBayes, Bayesian posterior probability (BPP) is indicated for each internal branch. The lineages found in this study are shown in blue, red, and pink. The blue stripe represents clade 2 with lineages related to *P. odocoilei* reported in ungulates host: *O. virginianus, O. bezoarticus*, *M. nemorivaga* y *M. americana.* The red stripe indicates clade 1, which consists of the *P. odocoilei*-related lineages present in the host species: *O. virginianus* and *M. americana*, and the pink lineage indicates the only *P. malariae/brasilianum* lineage found in *M. americana*. Host silhouettes in which the studied parasite lineages were found is shown. (For interpretation of the references to colour in this figure legend, the reader is referred to the web version of this article.)

Phylogenetic analysis of the partial *Plasmodium cytb* sequences from the brocket deer in the present study and sequences from North American *P. odocoilei* showed that 83.3% (5/6) of the sequences in the Amazonian brocket deer are related to North American ungulate parasite species. Sequences in two red-brocket deer were monophyletic with *P. odocoilei* clade 1 sequences obtained from North American white-tailed deer and *Anopheles* mosquitoes (with ML/BPP of 98/0.99, respectively), and sequences of three brocket deer (two red- and one gray-brocket deer) formed a monophyletic clade with *P. odocoilei* clade 2, with low support (54/0.96, respectively). Three, four and five nucleotides out of 979 bp (ref. MH177860) and 607 bp (ref. KU133755 and KU133748), separated the red-brocket deer *Plasmodium* sequences from the *P. odocoilei* clade 1 sequences, respectively. Up to three related *P. odocoilei* clade 2 strains co-circulated in the Amazonian-brocket deer population studied. One of the sequences in the red-brocket deer differed by three nucleotides out of 419 bp from the *P. odocoilei* allele D lineage (ref. MG709250). A three nucleotides difference from the *P. odocoilei* allele B lineage (ref. KU133750) out of 607 bp was also observed. The *Plasmodium* sp. sequence in the only positive gray-brocket deer differed by three nucleotides out of 607 bp amplified from the *P. odocoilei* allele E/F lineage (ref. KU133754). One red-brocket deer *Plasmodium* sp. sequence was identical to *Plasmodium malariae/brasilianum* (Fig. 2).

**Fig. 3.**
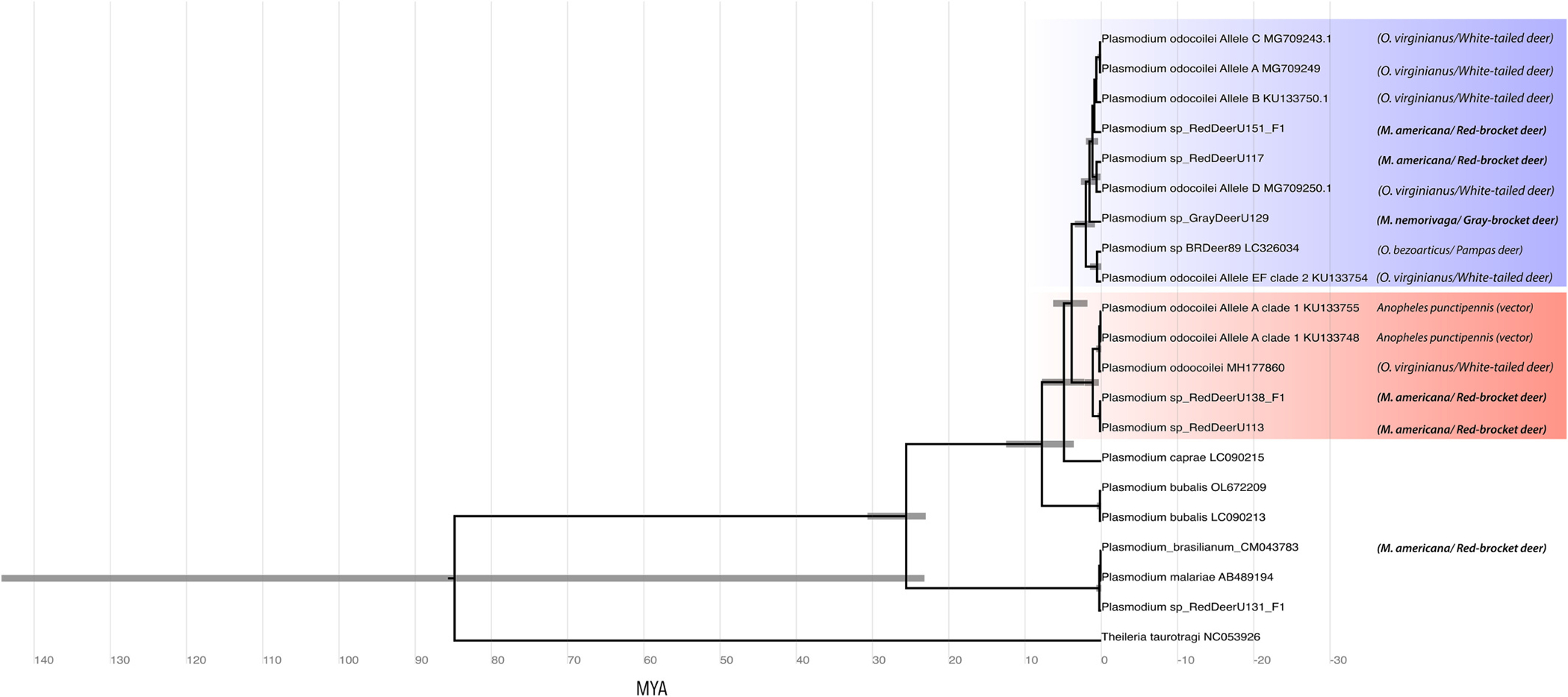
Time-calibrated phylogeny of maximum cladistic credibility based on mtDNA of *Plasmodium* species lineages from ungulates. TMRCA confidence intervals are shown at nodes. The blue stripe represents clade 2 with lineages related to *P. odocoilei* and the red stripe indicates clade 1 with lineages related to *P. odocoilei*. The reference lineages are those with accession numbers. Next to each lineage is the name of the host in which the parasite lineage was found. (For interpretation of the references to colour in this figure legend, the reader is referred to the web version of this article.)

Divergence estimates suggest *P. odocoilei* clade 1 and 2 lineages of the North and South American deer diverged approximately 2.6–3.5 MYA ago. The estimated divergence of lineages related to *P. odocoilei* clade 1 found in the red-brocket and the white-tailed deer was more recent (0.8–1.0 MYA), as was the time of divergence between *P. odocoilei* clade 2 lineages in the same host species (0.6–1.1 MYA). When other deer species were included, the divergence time estimate between *P. odocoilei* clade 2 lineages of the white-tailed deer and those in the Amazonian-brocket deer was 1.1–1.4 MYA, and in the Amazonian-brocket deer and the Brazilian pampas deer, 1.4–1.9 MYA (Fig. 3).

## 4. Discussion

The current study indicates that malaria parasite infection is frequent widespread in wild Amazonian-brocket deer (55.6%). These findings highlight features of the ecology of *Plasmodium* lineages closely related to *P. odocoilei* species that naturally infect Amazonian deer. First, studies suggested that these parasites are not host specific, as they can infect different species (Asada et al., 2018; Guggisberg et al., 2018; Martinsen et al., 2016; Templeton et al., 2016a). However, their host range is apparently restricted to New World deer, as the lineages have not been detected in any other ungulates. The parasites may have an ecogeographic range with multiple potential hosts, and therefore these *Plasmodium* spp. lineages are likely of concern in the Americas (Gutiérrez et al., 2017).

We report for the first time the presence of lineages related to clade 1 of *P. odocoilei* in South America, found in wild Amazonian red-brocket deer. In addition, three haplotypes related to clade 2 of *P. odocoilei* were identified in wild Amazonian red and gray-brocket deer. Until the present study, only lineages related to clade 2 of *P. odocoilei* had been described in South America in wild pampas deer (*O. bezoarticus*) from Brazilian Pantanal (Asada et al., 2018). These results are consistent with previous studies that identified up to seven different *cytb* alleles of *P. odocoilei* clade 2 in the white-tailed deer (*O. virginianus*) in North America(Guggisberg et al., 2018; Martinsen et al., 2016) and consistent with a monophyletic grouping of all ungulate malaria parasites (Templeton et al., 2016a).

The rate of divergence suggest that the *P. odocoilei* clade 1 and 2 lineages diverged approximately 2.6–3.5 MYA, a result consistent with that reported by Asada and collaborators (Asada et al., 2018). Therefore, one possible evolutionary scenario that would explain the existence of lineages related to both parasite clades in the American deer is that the divergence of these lineages occurred after American deer divergence from a common ancestor with other deer lineages (~5 MYA)(Duarte et al., 2008). The Great American Biotic Interchange - GABI (~3 MYA) between North America and South America (Carrillo et al., 2020; Domingo et al., 2020) may have been the event that separated these lineages and their parasites. However, due to the lack of fossil records and lack of reports of *Plasmodium* lineages from ungulates in South America it is currently not possible to test this hypothesis (Asada et al., 2018).

The estimated date of divergence of the parasitic lineages coincides with the invasion of at least eight distinct ancestral forms of land deer that migrated from North America to South America and have been grouped into two major clades: the gray clade, which includes the gray brocket (*Mazama gouazoubira* and *M. nemorivaga*), the marsh deer (*Blastocerus dichotomus*), the huemul (*Hippocamelus bisulcus*), the taruka (*Hippocamelus antisensis*), and the pampas deer (*O. bezoarticus*); and the red clade, which includes the species of the red deer group (*Mazama bororo, Mazama nana, Mazama temama* and *M. americana*) and the genus *Odocoileus* (Carrillo et al., 2020; Duarte et al., 2008). We hypothesize that the differentiation of *P. odocoilei* clade 1 lineages may have occurred in ancestral host species that gave rise to the “red clade” of American deer, which could indicate that these parasites have adapted to the red clade. However, the absence of infection of clade 1 lineages in “gray clade” hosts may reflect insufficient sampling and not necessarily the absence of the specific lineage in this group of ungulates.

An alternative hypothesis is that, given the longer divergence time of lineages within clade 2 relative to clade 1 of *P. odocoilei* (~1. 9 MYA), common ancestral parasite lineages of both clades in South America originated from clade 2 lineages found in a North American deer ancestor (Asada et al., 2018), possibly in separate transitions of hosts representing both the “red clade” and the “gray clade” of South American ungulates during the GABI. The radiation and adaptation of ungulates in the South American area could have led to the speciation of lineages belonging to both clades in South America. Furthermore, sequences found of the *Plasmodium* species in the “red clade” (*M. americana* and *O. virginianus*) show divergence within each of the clades (1 and 2) with a possible recent divergence (~1 MYA). This could reflect the separation of South American red deer during the Pleistocene (Domingo et al., 2020; Duarte et al., 2008), resulting in the isolation of these ungulates after their colonization of South America.

A sequence identical to *P. malariae/brasilianum*, was identified in red-brocket deer suggesting that ungulates are probably incidental hosts of other *Plasmodium* lineages, possibly through habitat sharing with other vertebrate hosts. However, further studies are needed to determine parasite host range (Chaves et al., 2022; Grigg and Snounou, 2017; Joyner et al., 2015; Silva et al., 2019). Even if only incidental hosts, it would be important to determine how often ungulates are sink hosts for simian malarias, given that frequent infections could facilitate the evolution of novel parasites in a group where there have been frequent host-shifts (Wolfe et al., 2007). The *Plasmodium* spp. lineages could be transmitted among different mammalian wildlife taxa by generalist vectors than by species with greater parasite specificity (Boundenga et al., 2016; Galen et al., 2018; Lutz et al., 2016). Such vector behavior has been proposed as an adaptive trait in response to temporal fluctuations in host diversity and density in forest environments such as Amazonia (Boundenga et al., 2016). However, this trait could increase the possibility of interspecific transfer of parasite lineages that have a propensity to infect different host species, which is a concern for global health.

## 5. Conclusions

We conclude that *Plasmodium* species from both clades 1 and 2 of *P. odocoilei* are present in Amazonian red-brocket deer. The phylogenetic history of *Plasmodium* lineages infecting South American deer may represent phylogenetic associations with their hosts. The estimated time of lineage divergence was 2.6–3.5 MYA, which coincides with the migration of artiodactyls from North to South America, and this event may have contributed to the presumed initial speciation of clade 1-related lineages and multiple clade 2-related lineages of the *P. odocoilei* parasite in South American deer. The possibility that ungulates are incidental hosts of other *Plasmodium* species cannot be excluded.

## Data Availability

The consensus sequences that were generated from the six Plasmodium spp. strains have been deposited in NCBI under accession numbers OR759077 - OR759082 (GenBank, SGA sequences). Registration and publication of the sample analysis protocol will be on Protocols.io: https://www.protocols.io/private/6653CE1D8DD611ECA1850A58A9FEAC02. Other data used to support the findings of this study are available from the corresponding authors upon reasonable request.

## References

[R1] Amir A, Cheong FW, de Silva JR, Liew JWK, Lau YL, 2018. *plasmodium knowlesi* malaria: current research perspectives. Infect. Drug Resist. 11, 1145–1155. 10.2147/IDR.S148664. 30127631 PMC6089103

[R2] Asada M, Takeda M, Tomas WM, Pellegrin A, de Oliveira CHS, Barbosa JD, da Silveira JAG, Braga ÉM, Kaneko O, 2018. Close relationship of plasmodium sequences detected from south American pampas deer (Ozotoceros bezoarticus) to plasmodium spp. in north American white-tailed deer. Int. J. Parasitol. Parasi. Wildl. 7, 44–47. 10.1016/j.ijppaw.2018.01.001. PMC5963126 29845014

[R3] Aysanoa E, Mayor P, Mendoza AP, Zariquiey CM, Morales EA, Pérez JG, Bowler M, Ventocilla JA, González C, Baldeviano GC, Lescano AG, 2017. Molecular epidemiology of Trypanosomatids and Trypanosoma cruzi in Primates from Peru. Ecohealth 14, 732–742. 10.1007/s10393-017-1271-8. 29098492 PMC5818207

[R4] Bernárdez-Rodriguez GF, Bowler M, Braga-Pereira F, McNaughton M, Mayor P, 2021. Conservation education promotes positive short- and medium-term changes in perceptions and attitudes towards a threatened primate species. Ethnobiol. Conservat. 10 10.15451/ec2021-09-10.31-1-16.

[R5] Boundenga L, Makanga B, Ollomo B, Gilabert A, Rougeron V, Mve-Ondo B, Arnathau C, Durand P, Moukodoum ND, Okouga A-P, Delicat-Loembet L, Yacka-Mouele L, Rahola N, Leroy E, Ba CT, Renaud F, Prugnolle F, Paupy C, 2016. Haemosporidian parasites of antelopes and other vertebrates from Gabon, Central Africa. PLoS One 11, e0148958. 10.1371/journal.pone.0148958. 26863304 PMC4749209

[R6] Cáceres L, Calzada JE, Gabster A, Young J, Márquez R, Torres R, Griffith M, 2017. Social representations of malaria in the Guna indigenous population of Comarca Guna de Madungandi, Panama. Malar. J. 16, 256. 10.1186/s12936-017-1899-4. 28619033 PMC5472999

[R7] Carrillo JD, Faurby S, Silvestro D, Zizka A, Jaramillo C, Bacon CD, Antonelli A, 2020. Disproportionate extinction of south American mammals drove the asymmetry of the great American biotic interchange. Proc. Natl. Acad. Sci. 117, 26281–26287. 10.1073/pnas.2009397117. 33020313 PMC7585031

[R8] Chaves BA, de Alvarenga DAM, de Pereira MOC, Gordo M, Da Silva EL, Costa ER, de Medeiros ASM, Pedrosa IJM, Brito D, Lima MT, Mourão MP, Monteiro WM, Vasilakis N, de Brito CFA, Melo GC, Lacerda MVG, 2022. Is zoonotic plasmodium vivax malaria an obstacle for disease elimination? Malar. J. 21, 343. 10.1186/s12936-022-04349-6. 36397077 PMC9673391

[R9] de Abreu FVS, dos Santos E, Mello ARL, Gomes LR, de Alvarenga DAM, Gomes MQ, Vargas WP, Bianco-Júnior C, de Pina-Costa A, Teixeira DS, Romano APM, de Manso PPA, Pelajo-Machado M, Brasil P, Daniel-Ribeiro CT, de Brito CFA, de Ferreira-da-Cruz MF, Lourenço-de-Oliveira R, 2019. Howler monkeys are the reservoir of malarial parasites causing zoonotic infections in the Atlantic forest of Rio de Janeiro. PLoS Negl. Trop. Dis. 13, e0007906 10.1371/journal.pntd.0007906. 31815937 PMC6922453

[R10] Domingo L, Tomassini RL, Montalvo CI, Sanz-Pérez D, Alberdi MT, 2020. The great American biotic interchange revisited: a new perspective from the stable isotope record of argentine pampas fossil mammals. Sci. Rep. 10, 1608. 10.1038/s41598-020-58575-6. 32005879 PMC6994648

[R11] Duarte JMB, González S, Maldonado JE, 2008. The surprising evolutionary history of south American deer. Mol. Phylogenet. Evol. 49, 17–22. 10.1016/j.ympev.2008.07.009. 18675919

[R12] Galen SC, Borner J, Martinsen ES, Schaer J, Austin CC, West CJ, Perkins SL, 2018. The polyphyly of *plasmodium* : comprehensive phylogenetic analyses of the malaria parasites (order Haemosporida) reveal widespread taxonomic conflict. R. Soc. Open Sci. 5, 171780 10.1098/rsos.171780. 29892372 PMC5990803

[R13] González-Olvera M, Hernandez-Colina A, Pérez JG, Ulloa GM, Montero S, Maguiña JL, Lescano AG, Santolalla ML, Baylis M, Mayor P, 2022. Haemosporidians from a neglected Group of Terrestrial Wild Birds in the Peruvian Amazonia. Ecohealth 19, 402–416. 10.1007/s10393-022-01612-9. 36030330 PMC9573858

[R14] Grigg MJ, Snounou G, 2017. Plasmodium simium : a Brazilian focus of anthropozoonotic vivax malaria? Lancet Glob. Health 5, e961–e962. 10.1016/S2214-109X(17)30343-1. 28867402

[R15] Guggisberg AM, Sayler KA, Wisely SM, Odom John AR, 2018. Natural History of *Plasmodium Odocoilei* Malaria Infection in Farmed White-Tailed Deer. mSphere 3. 10.1128/mSphere.00067-18. PMC5907657 29669881

[R16] Guindon S, Dufayard J-F, Lefort V, Anisimova M, Hordijk W, Gascuel O, 2010. New algorithms and methods to estimate maximum-likelihood phylogenies: assessing the performance of PhyML 3.0. Syst. Biol. 59, 307–321. 10.1093/sysbio/syq010. 20525638

[R17] Gutiérrez EE, Helgen KM, McDonough MM, Bauer F, Hawkins MTR, Escobedo-Morales LA, Patterson BD, Maldonado JE, 2017. A gene-tree test of the traditional taxonomy of American deer: the importance of voucher specimens, geographic data, and dense sampling. Zookeys 697, 87–131. 10.3897/zookeys.697.15124. PMC5673856 29134018

[R18] Huelsenbeck JP, Ronquist F, 2001. MRBAYES: Bayesian inference of phylogenetic trees. Bioinformatics 17, 754–755. 10.1093/bioinformatics/17.8.754. 11524383

[R19] Jacob M, Medeiros Souza A, Martins de Carvalho A, de Vasconcelos Alves, Neto CF, Tregidgo D, Hunter D, De Oliveira Pereira F, Ros Brull G, Kunhlein VH, Guedes da Silva LJ, Mont’Alverne Jucá Seabr L, de Drewinski MP, Menolli N Jr., Carignano Torres P, Mayor PFM, Lopes P, Vasconcelos da Silva RR, Marcelino Gomes S, Da Silva-Maia JK, 2023. Food biodiversity as an opportunity to address the challenge of improving human diets and food security. Ethnobiol. Conservat. 12 10.15451/ec2023-02-12.05-1-14.

[R20] Joyner C, Barnwell JW, Galinski MR, 2015. No more monkeying around: primate malaria model systems are key to understanding plasmodium vivax liver-stage biology, hypnozoites, and relapses. Front. Microbiol. 6 10.3389/fmicb.2015.00145. PMC4374475 25859242

[R21] Kaewthamasorn M, Takeda M, Saiwichai T, Gitaka JN, Tiawsirisup S, Imasato Y, Mossaad E, Sarani A, Kaewlamun W, Channumsin M, Chaiworakul S, Katepongpun W, Teeveerapunya S, Panthong J, Mureithi DK, Bawm S, Htun LL, Win MM, Ismail AA, Ibrahim AM, Suganuma K, Hakimi H, Nakao R, Katakura K, Asada M, Kaneko O, 2018. Genetic homogeneity of goat malaria parasites in Asia and Africa suggests their expansion with domestic goat host. Sci. Rep. 8, 5827. 10.1038/s41598-018-24048-0. 29643434 PMC5895593

[R22] Kearse M, Moir R, Wilson A, Stones-Havas S, Cheung M, Sturrock S, Buxton S, Cooper A, Markowitz S, Duran C, Thierer T, Ashton B, Meintjes P, Drummond A, 2012. Geneious basic: an integrated and extendable desktop software platform for the organization and analysis of sequence data. Bioinformatics 28, 1647–1649. 10.1093/bioinformatics/bts199. 22543367 PMC3371832

[R23] Lutz HL, Patterson BD, Kerbis Peterhans JC, Stanley WT, Webala PW, Gnoske TP, Hackett SJ, Stanhope MJ, 2016. Diverse sampling of east African haemosporidians reveals chiropteran origin of malaria parasites in primates and rodents. Mol. Phylogenet. Evol. 99, 7–15. 10.1016/j.ympev.2016.03.004. 26975691

[R24] Martinsen ES, Paperna I, Schall JJ, 2006. Morphological versus molecular identification of avian Haemosporidia: an exploration of three species concepts. Parasitology 133, 279–288. 10.1017/S0031182006000424. 16740182

[R25] Martinsen ES, McInerney N, Brightman H, Ferebee K, Walsh T, McShea WJ, Forrester TD, Ware L, Joyner PH, Perkins SL, Latch EK, Yabsley MJ, Schall JJ, Fleischer RC, 2016. Hidden in plain sight: cryptic and endemic malaria parasites in north American white-tailed deer (*Odocoileus virginianus*). Sci. Adv. 2 10.1126/sciadv.1501486. PMC4788485 26989785

[R26] Mayor P, Ventocilla JA, Bowler M, Baldeviano GC, Pérez-Velez ES, Lescano AG, Aysanoa E, Morales EA, Pérez J, 2017. Prevalence of Trypanosoma cruzi and other Trypanosomatids in frequently-hunted wild mammals from the Peruvian Amazon. Am. J. Trop. Med. Hyg. 97, 1482–1485. 10.4269/ajtmh.17-0028. 29140234 PMC5817741

[R27] Pacheco MA, Battistuzzi FU, Junge RE, Cornejo OE, Williams CV, Landau I, Rabetafika L, Snounou G, Jones-Engel L, Escalante AA, 2011. Timing the origin of human malarias: the lemur puzzle. BMC Evol. Biol. 11, 299. 10.1186/1471-2148-11-299. 21992100 PMC3228831

[R28] Perkins SL, Schaer J, 2016. A modern menagerie of mammalian malaria. Trends Parasitol. 32, 772–782. 10.1016/j.pt.2016.06.001. 27492115

[R29] Price MN, Dehal PS, Arkin AP, 2009. FastTree: computing large minimum evolution trees with profiles instead of a distance matrix. Mol. Biol. Evol. 26, 1641–1650. 10.1093/molbev/msp077. 19377059 PMC2693737

[R30] Silva JC, Egan A, Friedman R, Munro JB, Carlton JM, Hughes AL, 2011. Genome sequences reveal divergence times of malaria parasite lineages. Parasitology 138, 1737–1749. 10.1017/S0031182010001575. 21118608 PMC3081533

[R31] Silva TRM, Barros FNL, Bahia M, Sampaio Junior FD, Santos SSF, Inoue LS, Gonçalves TS, Chiesorin Neto L, Faria DCLO, Tochetto C, Viana GMR, Monteiro FOB, Góes-Cavalcante G, Scofield A, 2019. *Plasmodium vivax* and *plasmodium falciparum* infection in Neotropical primates in the western Amazon, Brazil. Zoonoses Public Health 66, 798–804. 10.1111/zph.12626. 31293103

[R32] Templeton TJ, Asada M, Jiratanh M, Ishikawa SA, Tiawsirisup S, Sivakumar T, Namangala B, Takeda M, Mohkaew K, Ngamjituea S, Inoue N, Sugimoto C, Inagaki Y, Suzuki Y, Yokoyama N, Kaewthamasorn M, Kaneko O, 2016a. Ungulate malaria parasites. Sci. Rep. 6, 23230. 10.1038/srep23230. 26996979 PMC4800408

[R33] Templeton TJ, Martinsen E, Kaewthamasorn M, Kaneko O, 2016b. The rediscovery of malaria parasites of ungulates. Parasitology 143, 1501–1508. 10.1017/S0031182016001141. 27444556

[R34] Thompson JD, Higgins DG, Gibson TJ, 1994. CLUSTAL W: improving the sensitivity of progressive multiple sequence alignment through sequence weighting, position-specific gap penalties and weight matrix choice. Nucleic Acids Res. 22, 4673–4680. 10.1093/nar/22.22.4673. 7984417 PMC308517

[R35] Voinson M, Nunn CL, Goldberg A, 2022. Primate malarias as a model for cross-species parasite transmission. Elife 11. 10.7554/eLife.69628. PMC8798051 35086643

[R36] Wolfe ND, Dunavan CP, Diamond J, 2007. Origins of major human infectious diseases. Nature 447, 279–283. 10.1038/nature05775. 17507975 PMC7095142

